# Supplementary material for: Exploration of the Effect of Blue Light on Functional Metabolite Accumulation in Longan Embryonic Calli via RNA Sequencing
Source: Int J Mol Sci. 2019 Jan 21;20(2):441. doi: 10.3390/ijms20020441 (PMC6359358; doi:10.3390/ijms20020441)
Supplement: Supplementary file 1 [file ijms-20-00441-s001.pdf]

Supplementary Material

Exploration of the Effect of Blue Light on Functional Metabolite Accumulation in Longan Embryonic Calli via RNA Sequencing

Hansheng Li [1,2], Yumeng Lyu [1], Xiaohui Chen [1], Congqiao Wang [1], Deheng Yao [1], Shanshan Ni [1], Yuling Lin [1], Yukun Chen [1], Zihao Zhang [1], Zhongxiong Lai [1,*]

Table S1. Carotenoid contents of longan ECs under different light qualities.

Light quality	Light intensity ($\mu mol \cdot m^{-2} \cdot s^{-1}$)	Photoperiod (h)	Carotenoid contents 1 ($ug \cdot g^{-1}$DW)	Carotenoid contents 2 ($ug \cdot g^{-1}$ DW)	Carotenoid contents 3 ($ug \cdot g^{-1}$ DW)	Average Carotenoid contents ($ug \cdot g^{-1}$ DW)	Standard deviation	Duncan (5%)
Dark	0		10.37	12.29	11.30	11.32	0.96	c
Blue	32	12	16.52	18.11	18.83	17.82	1.18	a
White	32	12	13.65	11.86	13.24	12.65	0.94	b

Table S2. The SOD activities in longan ECs under different light qualities

Light quality	Light intensity ($\mu mol \cdot m^{-2} \cdot s^{-1}$)	Photoperiod (h)	SOD contents 1 ($u \cdot g^{-1}$)	SOD contents 2 ($u \cdot g^{-1}$)	SOD contents 3 ($u \cdot g^{-1}$)	Average SOD contents ($u \cdot g^{-1}$)	Standard deviation	Duncan (5%)
Dark	0		4.76	5.31	5.49	5.19	0.38	c
Blue	32	12	18.69	26.85	25.95	23.83	4.47	a
White	32	12	14.00	9.63	15.51	13.04	3.05	b

Table S3. The POD activities in longan ECs under different light qualities

Light quality	Light intensity (μmol·m^{-2}·s^{-1})	Photoperiod (h)	POD contents 1 (u·g^{-1})	POD contents 2 (u·g^{-1})	POD contents 3 (u·g^{-1})	Average POD contents (u·g^{-1})	Standard deviation	Duncan (5%)
Dark	0		2860	3220	3480	3186.67	311.34	c
Blue	32	12	4060	3920	3860	3946.67	102.63	a
White	32	12	4080	3500	3220	3600.00	438.63	b

Table S4. The H_2O_2 contents in longan EC under different light qualities

Light quality	Light intensity (μmol·m^{-2}·s^{-1})	Photoperiod (h)	H_2O_2 contents 1 (umol·g^{-1})	H_2O_2 contents 2 (umol·g^{-1})	H_2O_2 contents 3 (umol·g^{-1})	Average H_2O_2 contents (umol·g^{-1})	Standard deviation	Duncan (5%)
Dark	0		6.62	4.07	9.30	6.66	2.61	c
Blue	32	12	21.76	28.60	27.93	26.09	3.77	a
White	32	12	16.00	21.49	23.91	20.47	4.05	b

Table S5. The MDA contents in longan ECs under different light qualities

Light quality	Light intensity (μmol·m^{-2}·s^{-1})	Photoperiod (h)	MDA contents 1 (nmol·g^{-1})	MDA contents 2 (nmol·g^{-1})	MDA contents 3 (nmol·g^{-1})	Average MDA contents (nmol·g^{-1})	Standard deviation	Duncan (5%)
Dark	0		1.213	1.006	1.238	1.152	0.127	a
Blue	32	12	1.109	1.264	1.058	1.144	0.107	a
White	32	12	1.109	1.006	0.955	1.023	0.079	a

Table S6. The Top 5 enriched GO term of biological process in DB

NO.	Gene ontology term	Genome frequency of use	Corrected P-value
1	transmembrane transport	4.5%	6.95e-07
2	calcium ion transport	0.3%	0.00343
3	single-organism process	34.8%	0.01319
4	ion transport	4.4%	0.02260
5	phosphorylation	6.3%	0.05751

Table S7. The Top 5 enriched GO term of biological process in DW

NO.	Gene ontology term	Genome frequency of use	Corrected P-value
1	calcium ion transport	0.3%	0.22912
2	cell recognition	0.2%	0.50080
3	amine transport	0.1%	0.62262
4	carboxylic acid transmembrane transport	0.2%	0.89953
5	organic acid transmembrane transport	0.2%	1

Table S8. The Top 5 enriched GO term of cellular component in DB

NO.	Gene ontology term	Genome frequency of use	Corrected P-value
1	integral component of membrane	2%	4.65e-10
2	membrane	47.9%	1.00e-06
3	intrinsic component of membrane	34.1%	0.00017
4	membrane part	37.2%	0.00074
5	myosin complex	0.2%	0.00558

Table S9. The Top 5 enriched GO term of cellular component in DW

NO.	Gene ontology term	Genome frequency of use	Corrected P-value
1	external encapsulating structure	2.4%	0.03371
2	extracellular region	2.0%	0.55388
3	cell periphery	6.8%	1
4	Pex17p-Pex14p docking complex	0.1%	1
5	peroxisomal importomer complex	0.1%	1

Table S10. The Top 5 enriched GO term of molecular function in DB

NO.	Gene ontology term	Genome frequency of use	Corrected P-value
1	ATP binding	8.7%	1.60e-14
2	anion binding	11.7%	3.21e-14
3	purine nucleoside binding	9.9%	2.78e-13
4	purine ribonucleoside binding	9.9%	2.78e-13
5	nucleoside binding	10.0%	7.40e-13

Table S11. The Top 5 enriched GO term of molecular function in DW

NO.	Gene ontology term	Genome frequency of use	Corrected P-value
1	organic acid transmembrane transporter activity	0.2%	0.10172
2	oxidoreductase activity, acting on X-H and Y-H to form an X-Y bond	0.1%	0.24186
3	oxidoreductase activity, acting on X-H and Y-H to form an X-Y bond, with oxygen as acceptor	0.1%	0.24186
4	organic anion transmembrane transporter activity	0.3%	0.25920
5	NADPH dehydrogenase activity	0.0%	0.38483

Table S12. Primers information used for real-time PCR analysis of longan genes

Gene name	Primer sequences(5'→3')	Size(bp)	TM(°C)
CRY1-QF	TGATGCTCTTGGTTGGCAGTA	157	60
CRY1-QR	TCCATTCAGTTGGTAGTCTGGC		
CRY2-QF	TGGGCTTAGAGAATACTCCCG	163	60
CRY2-QR	TGGGCTTAGAGAATACTCCCG		
COP1-QF	ACGGCGTGTGGACATAGTTT	221	60
COP1-QR	AACTGACACCTCACAACCCTG		
HY5-QF	TGAACAGGAGCGAGTCTGAGT	188	60
HY5-QR	CAGAATCCAGAGGAGGACCA		
MYC2-QF	TTCGCTTTGGAGAGCCTATG	237	60
MYC2-QR	GAATCCCATTGCTGAAATCG		
PIF4-QF	GGGAACAAGTCAGCACAAAGA	223	60
PIF4-QR	CTGCCCATCCACATTACCTG		
SPA1-QF	TGTTGTGTCCAGTTTCCCTTG	195	60
SPA1-QR	GCTTCAATGTGTTATCCGTGG		
CIB1-QF	GCATACCTCCAGTTCAATCCAG	126	60
CIB1-QR	AGTAAGATGAGCCGAGGAACG		
CO-QF	CGTGTTCTTCAAGCAGCAGTAG	150	60
CO-QR	CAAGTATTCGCTTCCTCATTGG		
